# A novel autophagy-related long non-coding RNAs signature predicting progression-free interval and I-131 therapy benefits in papillary thyroid carcinoma

**DOI:** 10.1515/med-2023-0660

**Published:** 2023-03-03

**Authors:** Jie Hao, Shoujun Wang, Jinmiao Wang, Zhendong Zhang, Ming Gao, Yajuan Wan

**Affiliations:** Department of Breast and Thyroid Surgery, Tianjin Union Medical Center of Nankai University, Tianjin, 300121, PR China; Tianjin Key Laboratory of General Surgery in Construction, Tianjin Union Medical Center, Tianjin, PR China; College of Life Science, Nankai University, 94 Weijin Road, Nankai District, Tianjin, 300100, PR China; Department of Breast and Thyroid Surgery, Tianjin Union Medical Center of Nankai University, 190 Jie-Yuan Road, Hongqiao District, Tianjin, 300121, PR China

**Keywords:** papillary thyroid carcinoma, autophagy, lncRNAs, progression-free interval, I-131 therapy

## Abstract

This study aimed to explore the prognostic and predictive value of autophagy-related lncRNAs in papillary thyroid carcinoma (PTC). The expression data of autophagy-related genes and lncRNAs of the PTC patients were obtained from TCGA database. Autophagy-related-differentially expressed lncRNAs (DElncs) were identified and used to establish the lncRNAs signature predicting patients’ progression-free interval (PFI) in the training cohort. Its performance was assessed in the training cohort, validation cohort, and entire cohort. Effects of the signature on I-131 therapy were also explored. We identified 199 autophagy-related-DElncs and constructed a novel six-lncRNAs signature was constructed based on these lncRNAs. This signature had a good predictive performance and was superior to TNM stages and previous clinical risk scores. I-131 therapy was found to be associated with favorable prognosis in patients with high-risk scores but not those with low-risk scores. Gene set enrichment analysis suggested that a series of hallmark gene sets were enriched in the high-risk subgroup. Single-cell RNA sequencing analysis suggested that the lncRNAs were mainly expressed in thyroid cells but not stromal cells. In conclusion, our study constructed a well-performed six-lncRNAs signature to predict PFI and I-131 therapy benefits in PTC.

## 1 Introduction

The incidence of thyroid cancers (TC), the most frequent endocrine tumors, has been increasing during the past decades [1,2], largely due to the progressively available and sensitive use of diagnostic technologies [3,4]. Differentiated thyroid cancer (DTC), originating from follicular epithelial cells [5], accounts for over 95% of all TC. Most DTC is papillary thyroid cancer (PTC; 85–90%) [6,7]. PTC typically responds well to standard therapy, including radical surgery, radioactive iodine (I-131) therapy, and endocrine therapy, and has a relatively good prognosis with a more than 90% 10-year survival rate. However, some patients experience recurrence after initial treatment [8]. Therefore, it is crucial to develop novel biomarkers or risk models to accurately evaluate the prognosis of PTC to ensure patients with low risk are not over-treated while those with high risk receive appropriate aggressive treatment.

Autophagy, a critical intracellular process, degrades or removes damaged or denatured proteins and dysfunctional organelles in lysosomes and is essential to maintain cellular homeostasis, metabolism, and survival [9,10]. Abnormal autophagy is involved in various diseases and associated with cancer occurrence, development, and metastasis although its definitive role in carcinogenesis and underlying mechanisms are inconclusive [11]. It plays either a protective role by inhibiting tumor development in the early stages or a detrimental role by promoting tumor progression in advanced stages of cancers. Additionally, autophagy can enhance tumor resistance to chemotherapy or radiotherapy [12]. In thyroid cancer, some studies have revealed autophagy can regulate tumor development and dedifferentiation and is involved in drug resistance. Kim et al. found autophagy-related proteins, LC3A, LC3B, p62, and BNIP-3, differ according to thyroid cancer subtypes [13]. Plantinga et al. found autophagy activity is associated with clinical response to radioiodine therapy potentially via maintaining tumor cell differentiation in non-medullary thyroid cancer [14]. Wang et al. found combining vemurafenib and autophagy inhibitors exerts more pronounced tumor suppression in thyroid cancer [15]. Tesselaar et al. found that digitalis-like compounds, the autophagy activators, can restore human sodium-iodide symporter (hNIS) expression and iodide uptake in thyroid cancer cells and may be a promising strategy to overcome radioactive iodide resistance [16].

Long non-coding RNAs (lncRNAs) function in a series of cellular processes in cancers such as cell proliferation, autophagy, and genomic stability by regulating gene expression via diverse mechanisms [17]. The role of lncRNAs in thyroid cancer has been revealed gradually. In thyroid cancer, previous studies have revealed that some lncRNAs are associated with cell proliferation, apoptosis, and autophagy [18]. For example, lncRNA OIP5-AS1, regulated by METTL14, can promote PTC progression by miR-98/ADAMTS8 signaling [19]. LncRNA FER1L4 can promote PTC malignancy by targeting miR-612/CDH4 axis [20]. LncRNA MIAT can promote PTC invasion via miR-150/EZH2 pathway [21]. LncRNA TNRC6C-AS1 can inhibit cell proliferation and promote apoptosis and autophagy via Hippo signaling pathway in thyroid cancer cells [22]. LncRNA MALAT1 knockdown can inhibit tumor migration and invasion while increasing autophagy via miR-200a-3p/FOXA1 axis in thyroid cancer cells [23]. Considering the molecular and clinical value of autophagy and lncRNAs in thyroid cancer, we here aimed to establish an effective autophagy-related lncRNAs risk signature to predict progression-free interval (PFI) in PTC patients.

## 2 Methods and materials

### 2.1 Data collection and processing

**Figure 1:**
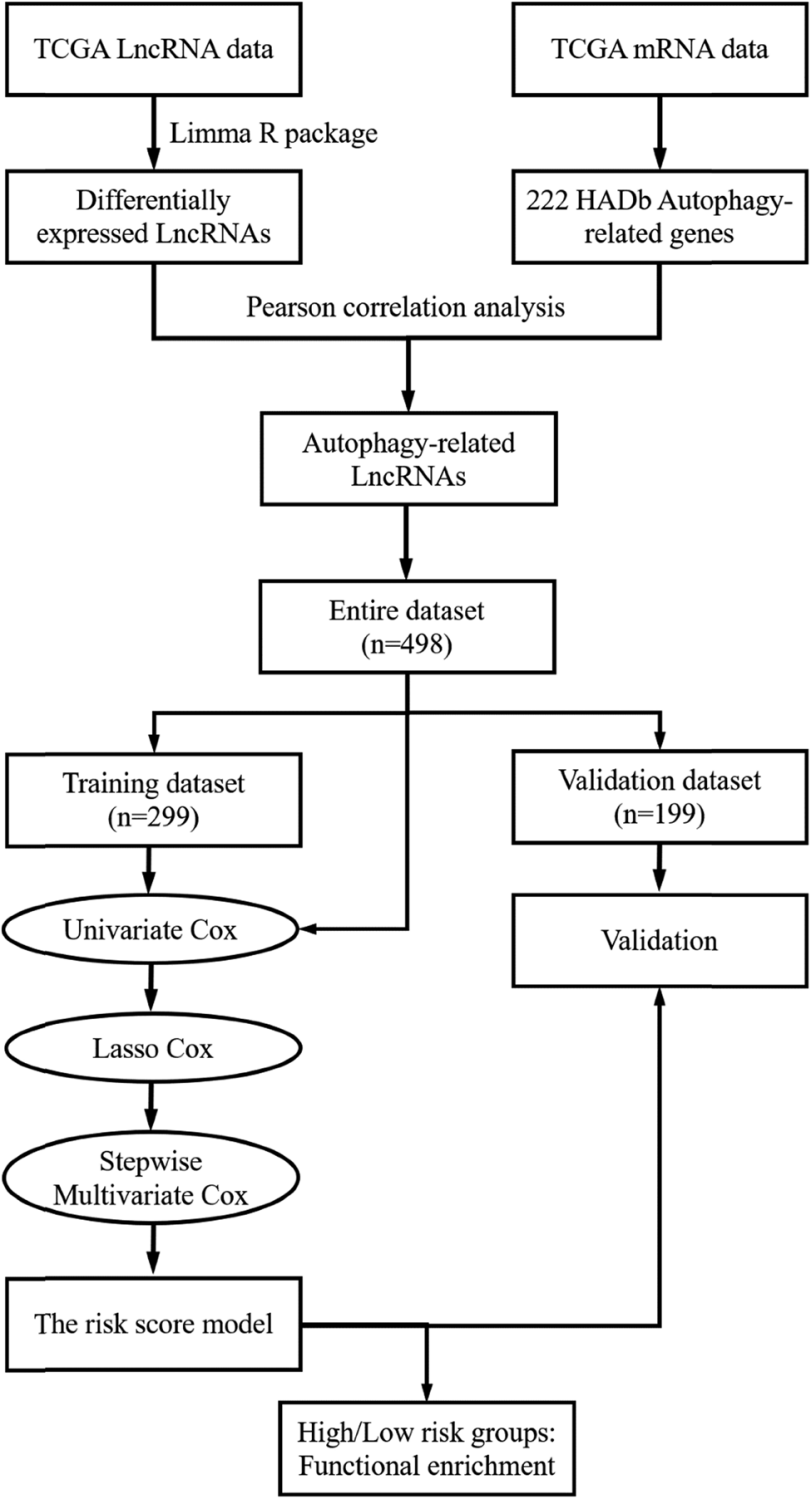
Flowchart.

The workflow of this study is presented in Figure 1. The expression data including mRNA and lncRNA of PTC patients were got from TCGA database (https://www.cbioportal.org) [24]. This dataset consisted of 507 subjects, 510 tumor samples, and 58 adjacent normal tissue samples. Only the patients with clinical data and follow-up time/PFI ≥ 30 days were included. Overall, 498 cases were finally included and were randomly split into a training cohort (*n* = 299) and a validation cohort (*n* = 199). A total 222 autophagy-related genes (ARGs) were obtained from the Human Autophagy Database (HADb, http://autophagy.lu/clustering/index.html), which collected those genes from the literature. LncRNAs and ARGs mRNAs expression were extracted according to GENCODE annotations (https://www.gencodegenes.org). LncRNAs with zero expression levels in more than 50% of samples were excluded. Clinical variables including age, gender, race, cancer history, thyroid gland disorder history, histological types, TNM stages, T stage, N stage, M stage, tumor location, residual tumor, American Thyroid Association (ATA) risk stratification, the distant metastasis, patient age, completeness of resection, local invasion, and tumor size (MACIS) scores were collected. The methods of assessing the tumors with ATA risk stratification and MACIS scores have been described in the previous report [24] and are introduced in the Supplementary data. We also re-evaluated the tumors based on the methods. Additionally, BRAF mutation and telomerase reverse transcriptase (TERT) promoter mutation status information were extracted, which have been reported to be associated with prognosis in TC [25]. Patients’ progression-free survival (PFS) and overall survival (OS) were also extracted.

### 2.2 Screening of the differentially expressed ARGs-related lncRNAs

We used the “Limma” package to identify the differentially expressed lncRNAs (DElncs) between tumor samples and control samples with the thresholds of fold change (FC) >1.5 or <1/1.5 and false discovery rate (FDR) <0.05 [26]. Subsequently, the correlation coefficient (*R* ^2^) between ARGs and DElncs was calculated by Pearson correlation analyses. The lncRNAs with *R* ^2^ > 0.25 and *P* < 0.001 were defined as autophagy-related lncRNAs.

### 2.3 Construction of autophagy-related lncRNAs signature

Univariate Cox regression analyses were performed to explore the associations of the autophagy-related DElncs with PFI. The autophagy-related DElncs that were associated with PFI in both the entire cohort and the training cohort (*P* < 0.1) were used as candidates to build the prognostic signature in the training cohort. The candidate DElncs were imputed into LASSO Cox regression analysis. The core autophagy-related DElncs tightly related to PFI were obtained when the optimal lambda value was achieved. The selected lncRNAs were then subjected to stepwise multivariate Cox regression analysis to build the autophagy-related lncRNAs risk signature. Each patient’s risk score was calculated by a linear combination of multiplying each lncRNA expression and the corresponding Cox regression coefficient. The patients were stratified into high-risk and low-risk groups by the median value. The PFI difference between the two groups was compared by Kaplan–Meier curves. Multivariate Cox regression analysis was performed to evaluate the independent prognostic value of the risk scores by adjusting the potential confounders. For the adjusted model I, the confounders was selected if they changed the effect estimate of the risk scores on PFI by more than 10% or were significantly associated with PFI. For the adjusted model II, the confounders in the adjusted model I and the remaining demographic data were adjusted. In addition, the area under the time-dependent ROC curves (AUC), as well as Harrell’s concordance index (C-index), were utilized to assess the predictive value of the autophagy-related lncRNAs risk signature. Validation was performed in the validation cohort and the entire cohort. The predictive performance of the risk model was also compared with TNM stages, ATA risk stratification, and MACIS scores by comparing their C-indices in the patients without missing values.

### 2.4 Association of the risk score with I-131 therapy efficacy

The association of the risk score with I-131 therapy efficacy was also explored with PFI as the primary endpoint and PFS and OS as the secondary endpoints. The patients were first divided into high and low risk with the optimal cutoff value, which was obtained based on the minimum *P*-value of the interaction test in univariate Cox analysis with PFI as the primary endpoint. I-131 therapy efficacy was investigated in patients with high or low risk by Kaplan–Meier curves with log-rank tests.

### 2.5 Gene set enrichment analysis (GSEA)

GSEA (version 3.0) was executed to assess the significantly different hallmark gene sets between different risk subgroups. The enriched gene sets whose/normalized enrichment score (NES)/> 1, nominal *P*-value <0.05, and FDR *q*-value <0.05 were treated as significant.

### 2.6 Single-cell RNA sequencing data analysis

The scRNA-seq data of 22 fresh surgical samples from six primary PTC tumors, six paired adjacent normal tissues, eight metastatic lymph nodes (LNs, including three recurrent LNs), and two subcutaneous metastatic loci were extracted from the GSE184362 dataset stored in the Gene Expression Omnibus (GEO) database [27]. Further analyses were performed with the R package, “Seurat,” using the standard data analysis pipeline [28]. Briefly, cells with low quality (the proportion of mitochondrial genes counts >10%, UMIs <500 or >5,000) were first removed; then the cell gene expression matrix was normalized and scaled with the default parameters; subsequently, the top 2,000 highly variable genes were identified by the FindVariableFeatures() function for the principal component analysis; fourth, the functions, FindNeighbors() and FindClusters() were utilized for cell clustering at a resolution of 0.4; next, the uniform manifold approximation and projection (UMAP) were used for visualization; cell markers provided in the previous report were used to annotate the cell clusters [27]. The cells with positive expression of lncRNAs of interest were assigned to the positive group and the other cells to the negative group. The differentially expressed genes between the two groups were identified through the FindAllMarkers() function, ranked by the log FC, and subjected to GSEA to explore the potential mechanisms.

### 2.7 Statistical analysis

R software v3.4.3 was utilized to perform all statistical analyses. The categorical variables were compared by chi-square tests or Fisher’s exact tests while the continuous variables were compared by Wilcox tests or Kruskal–Wallis tests. Kaplan–Meier curves were plotted to assess the prognosis differences between different groups stratified by risk scores and I-131 therapy efficacy. Cox regression analyses were carried out to identify lncRNAs that were associated with PFI. *P* < 0.05 was regarded as statistically significant unless otherwise stated.


**Ethics statement:** The current study has been approved by the Institutional Review Board of Tianjin Union Medical Center of Nankai University (2021-B34).

## 3 Results

### 3.1 Identification of differentially expressed autophagy-related lncRNAs

**Figure 2:**
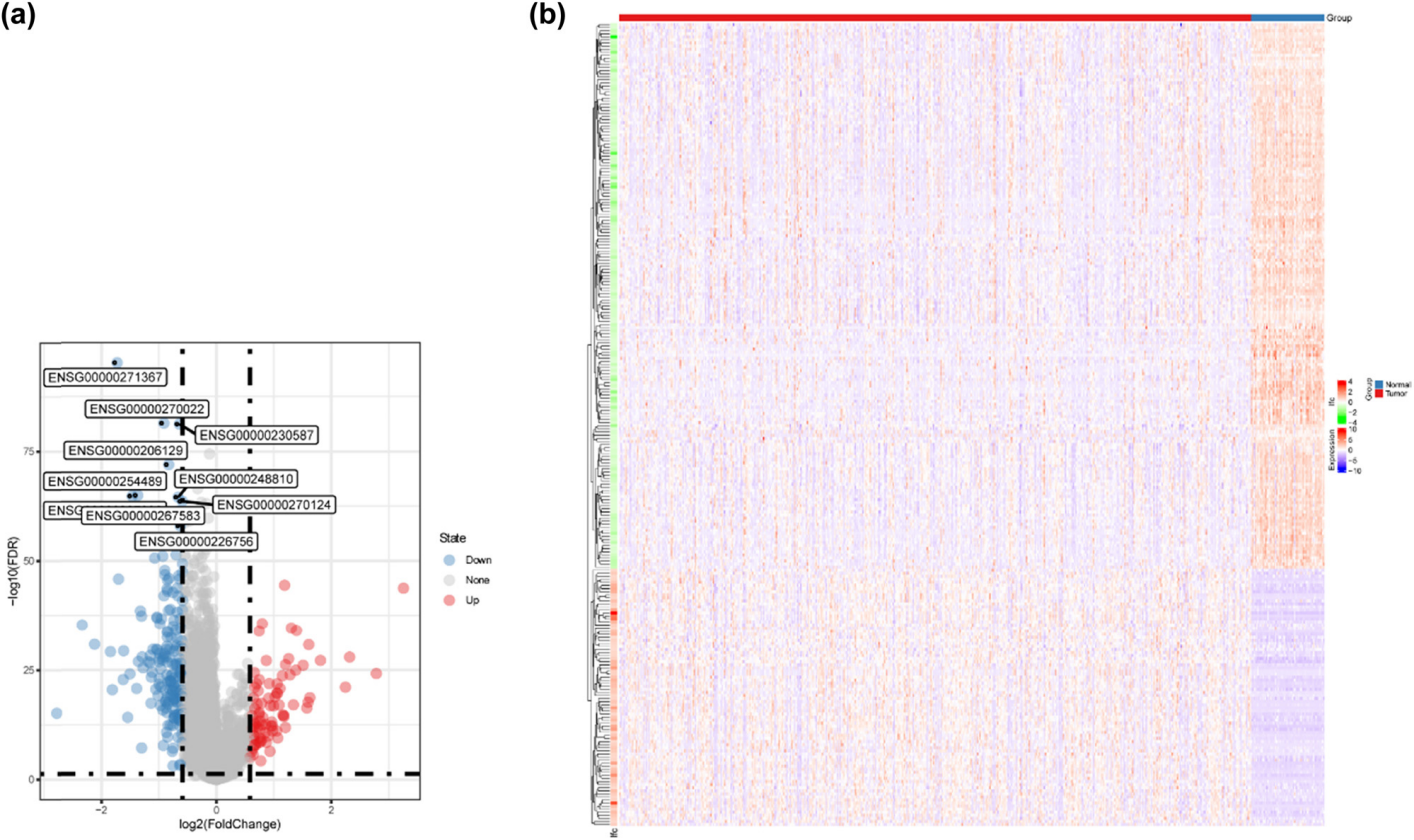
Comparison of lncRNAs expression in PTC tissues with adjacent tissues. (a) Volcano plot. The top ten DElncs were indicated based on the FDR, which were all downregulated. (b) Heatmap plot. Note: lfc, log2(FC).

The mRNA expression data of PTC tissues and adjacent normal tissues were obtained from the TCGA database. A total of 222 ARGs were generated from the HADb database and their expression data were extracted. About 14,822 lncRNAs were also extracted and 7,425 lncRNAs whose expressions were non-zero in more than 50% of samples were finally included. DElncs were identified between PTC and normal thyroid tissues via the “Limma” package. Based on the cutoff criteria, 262 DElncs with 178 lncRNAs downregulated and 84 lncRNAs upregulated were found. The volcano plot and heatmap for these 262 DElncs in tumor and adjacent tissues are displayed in Figure 2. Pearson correlation analyses were performed between these lncRNAs and 222 ARGs. Finally, 199 autophagy-related DElncs were attained based on the criteria of *R* ^2^ > 0.25 and *P* < 0.001.

### 3.2 Establishment of autophagy-related lncRNA risk signature

**Figure 3:**
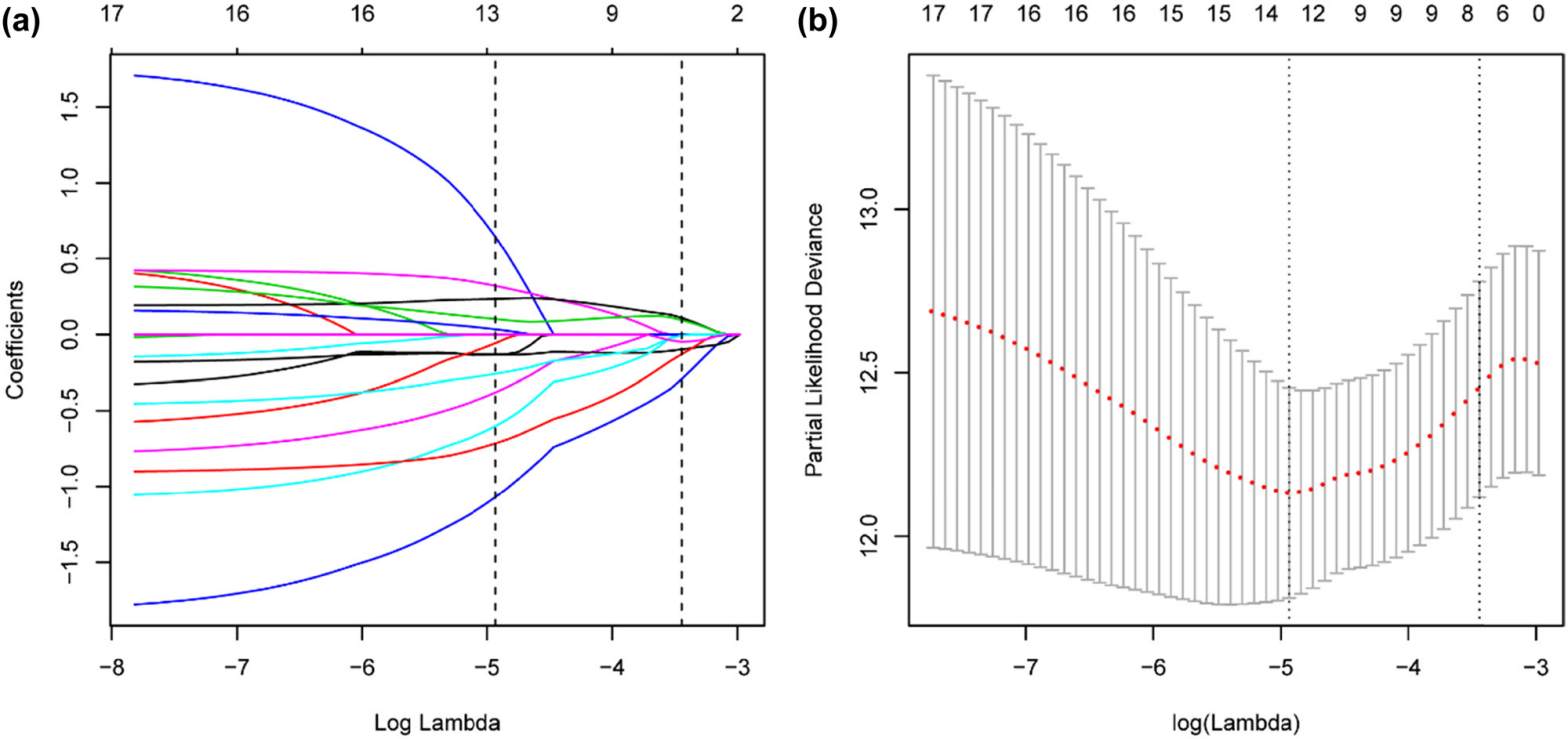
Screening of the core autophagy-related lncRNAs associated with PFI by LASSO COX regression analysis. (a) LASSO coefficient of the lncRNAs by 10-fold cross-validation. (b) Partial likelihood deviance with corresponding log(*λ*) values at the minimal deviance.

**Figure 4:**
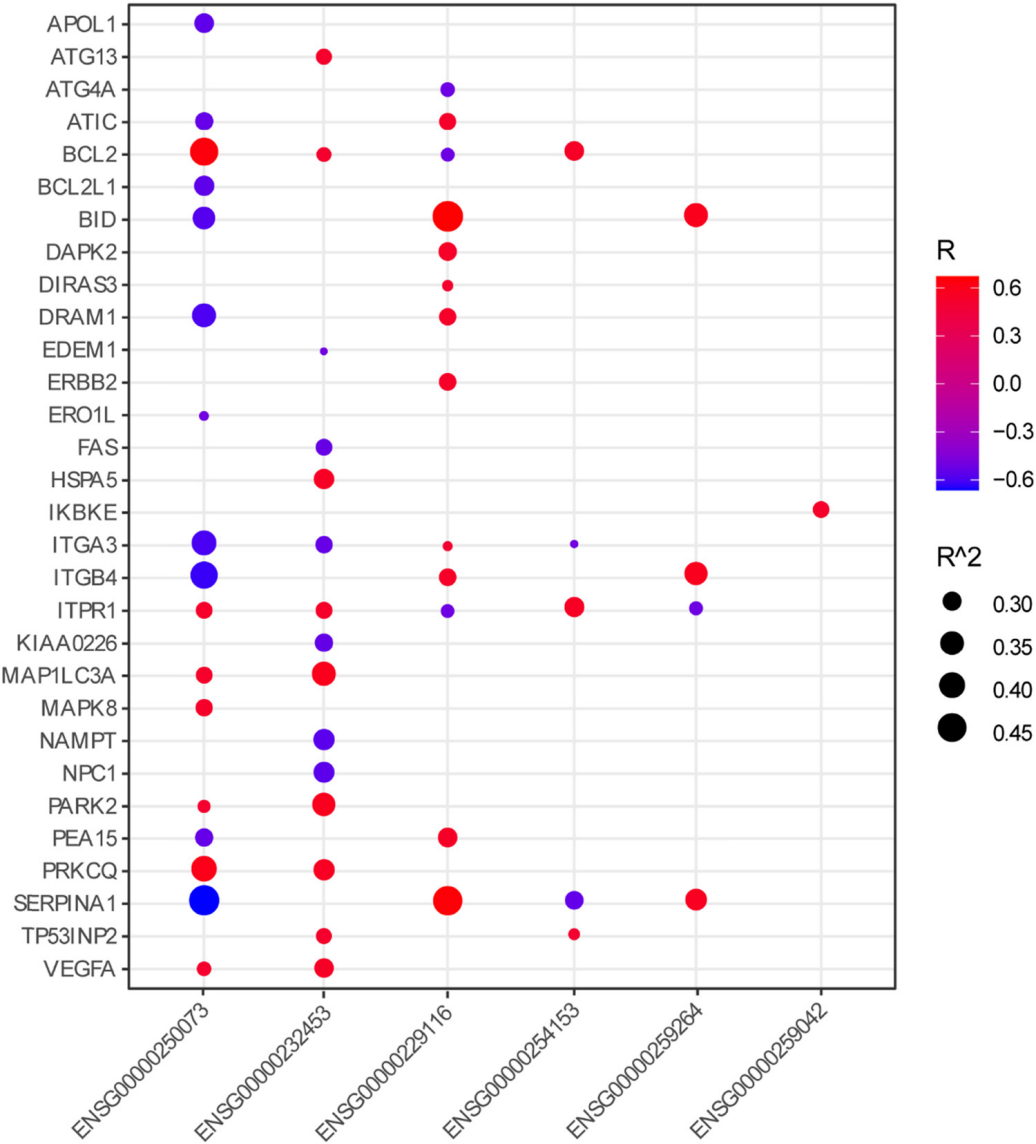
Correlation of the selected six lncRNAs with ARGs.

Four hundred ninety-eight PTC patients were included and randomly divided into a training cohort (*n* = 299) and a validation cohort (*n* = 199). The clinical characteristics of the two cohorts were similar (Table 1). Univariate Cox regression analyses were performed to explore the associations of the autophagy-related DElncs with PFI in the entire cohort and training cohort. The autophagy-related DElncs that were associated with PFI in both the entire and training cohort (*P* < 0.1) were imputed into LASSO Cox regression analysis in the training cohort. Thirteen core lncRNAs tightly related to PFI were obtained at optimal lambda value (Figure 3). The selected lncRNAs were then subjected to stepwise multivariate Cox regression analysis. Finally, an autophagy-related lncRNAs risk signature consisting of six lncRNAs was constructed by multiplying each lncRNA expression and the corresponding Cox regression coefficient (Table 2). The six lncRNAs were co-expressed with 31 ARGs (*R* ^2^ > 0.25; Figure 4).

**Table 1:** Comparison of the clinical characteristics between the training and validation cohorts

Cohort	Training	Validation	*P*-value
*N*	299	199	
Age (years)	47.0 ± 15.4	48.0 ± 16.4	0.483
MACIS scores (*N* = 486, unknown = 12)	5.4 ± 1.5	5.4 ± 1.6	0.585
**Gender**			0.991
Female	218 (72.9%)	145 (72.9%)	
Male	81 (27.1%)	54 (27.1%)	
**Race**			0.553
White	197 (65.9%)	132 (66.3%)	
Asian	35 (11.7%)	16 (8.0%)	
Others	16 (5.4%)	12 (6.0%)	
Unknown	51 (17.1%)	39 (19.6%)	
**Cancer history**			0.832
No	278 (93.0%)	186 (93.5%)	
Yes	21 (7.0%)	13 (6.5%)	
**Thyroid gland disorder history**			0.489
No	162 (54.2%)	113 (56.8%)	
Yes	98 (32.8%)	67 (33.7%)	
Unknown	39 (13.0%)	19 (9.5%)	
**Histological types**			0.838
Classical/usual	211 (70.6%)	141 (70.9%)	
Follicular	59 (19.7%)	42 (21.1%)	
Tall cell	24 (8.0%)	12 (6.0%)	
Others	5 (1.7%)	4 (2.0%)	
**TNM stage**			0.770
Stage I	168 (56.2%)	111 (55.8%)	
Stage II	29 (9.7%)	23 (11.6%)	
Stage III	68 (22.7%)	43 (21.6%)	
Stage IV	32 (10.7%)	22 (11.1%)	
Unknown	2 (0.7%)	0 (0.0%)	
**T stage**			0.867
T1	86 (28.8%)	56 (28.1%)	
T2	93 (31.1%)	69 (34.7%)	
T3	104 (34.8%)	66 (33.2%)	
T4	15 (5.0%)	7 (3.5%)	
TX	1 (0.3%)	1 (0.5%)	
**N stage**			0.245
N0	133 (44.5%)	95 (47.7%)	
N1	140 (46.8%)	80 (40.2%)	
NX	26 (8.7%)	24 (12.1%)	
**M stage**			0.773
M0	172 (57.5%)	109 (54.8%)	
M1	5 (1.7%)	4 (2.0%)	
MX	121 (40.5%)	86 (43.2%)	
Unknown	1 (0.3%)	0 (0.0%)	
**Tumor location**			0.921
Left lobe	109 (36.5%)	65 (32.7%)	
Right lobe	123 (41.1%)	87 (43.7%)	
Bilateral	50 (16.7%)	36 (18.1%)	
Isthmus	13 (4.3%)	9 (4.5%)	
Unknown	4 (1.3%)	2 (1.0%)	
**Residual tumor**			0.198
R0	226 (75.6%)	154 (77.4%)	
R1	38 (12.7%)	14 (7.0%)	
R2	3 (1.0%)	1 (0.5%)	
RX	16 (5.4%)	14 (7.0%)	
Unknown	16 (5.4%)	16 (8.0%)	
**ATA risk stratification**			0.166
Low	97 (32.4%)	73 (36.7%)	
Intermediate	179 (59.9%)	112 (56.3%)	
High	20 (6.7%)	8 (4.0%)	
Unknown	3 (1.0%)	6 (3.0%)	
**BRAF mutation**			0.191
No	115 (38.5%)	82 (41.2%)	
Yes	178 (59.5%)	108 (54.3%)	
Unknown	6 (2.0%)	9 (4.5%)	
**TERT mutation**			0.862
No	202 (67.6%)	138 (69.3%)	
Yes	23 (7.7%)	13 (6.5%)	
Unknown	74 (24.7%)	48 (24.1%)	

Note: ATA, American Thyroid Association.

**Table 2:** Information of the lncRNAs in the autophagy-related lncRNAs risk model for predicting PFI of patients with papillary thyroid carcinoma

ENSG ID	Symbol	Chromosome	Gene start (bp)	Gene end (bp)	Coefficient	*P*-value
ENSG00000254153	CTA-398F10.2	8	8456909	8461337	−1.892	0.002
ENSG00000232453	RP4-794H19.1	1	58882868	58931897	1.178	0.015
ENSG00000250073	RP11-677M14.3	11	124759129	124766119	−1.381	0.009
ENSG00000259264	RP11-60L3.1	15	74202705	74221555	0.440	0.037
ENSG00000229116	RP11-20J15.3	10	44282489	44293998	−0.840	0.003
ENSG00000259042	AE000661.50	14	22415362	22418657	−0.544	0.074

### 3.3 Validation of the autophagy-related lncRNA risk signature

**Figure 5:**
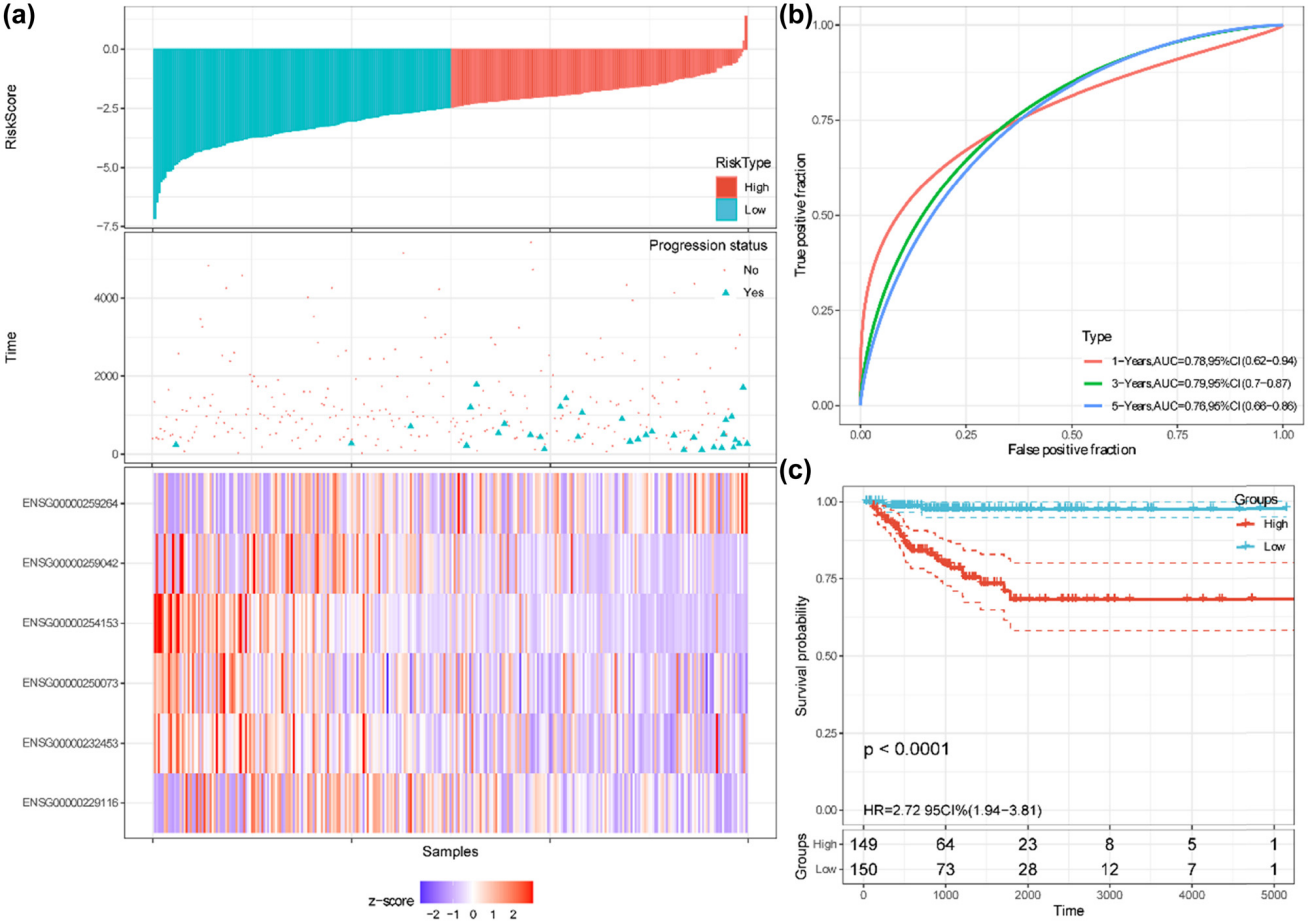
Predictive performance of the six autophagy-related lncRNAs signature in the training cohort. (a) Risk scores, PFI/progression status, and expression heatmap. (b) Time-dependent ROC curves for predicting 1-, 3-, and 5-year PFI based on the risk scores. (c) Kaplan–Meier curves of the patients with high and low-risk scores, which were divided by the median value.

**Figure 6:**
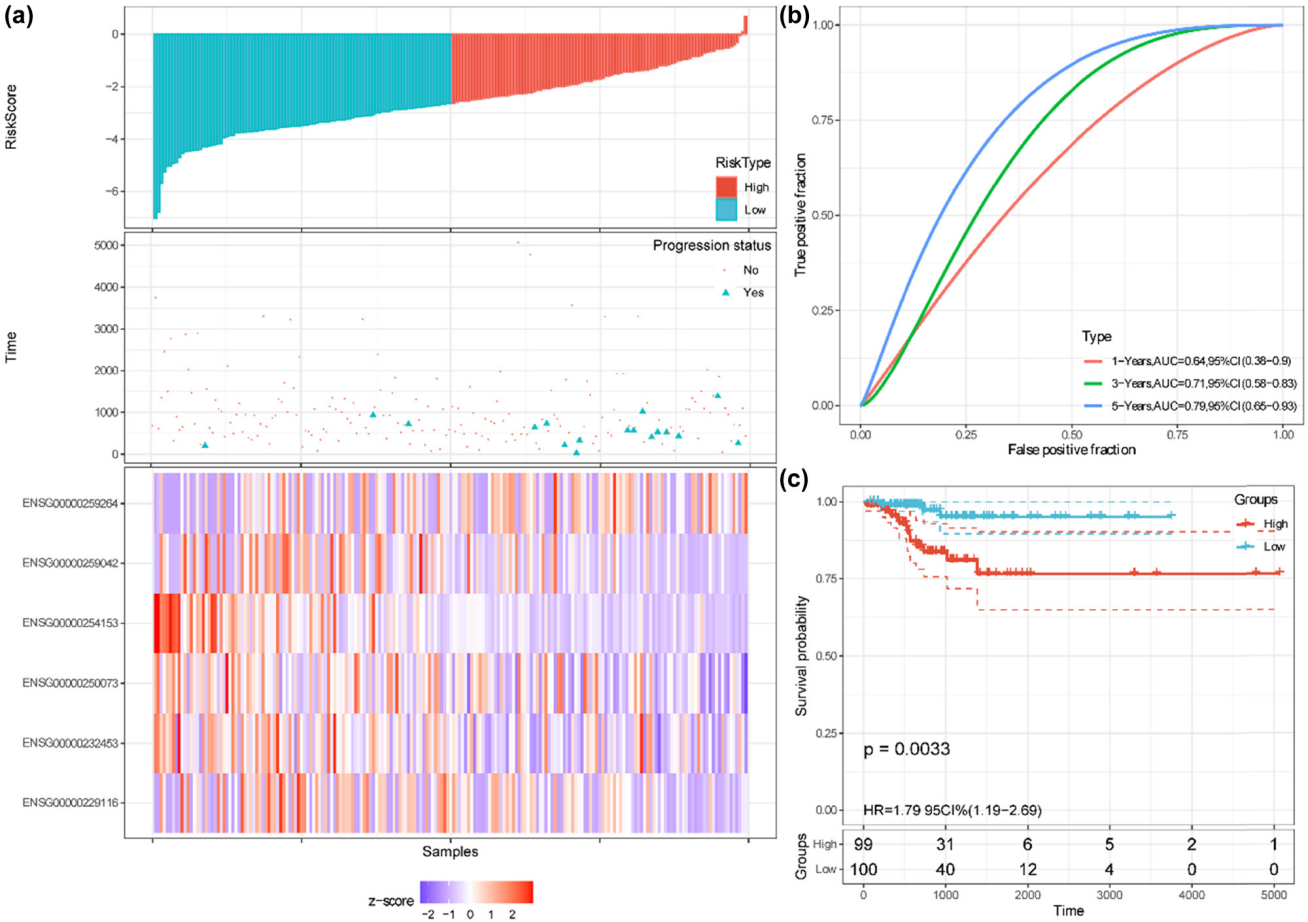
Predictive performance of the six autophagy-related lncRNAs signature in the validation cohort. (a) Risk scores, PFI/progression status, and expression heatmap. (b) Time-dependent ROC curves for predicting 1-, 3-, and 5-year PFI based on the risk scores. (c) Kaplan–Meier curves of the patients with high and low-risk scores, which were divided by the median value of the risk scores.

**Figure 7:**
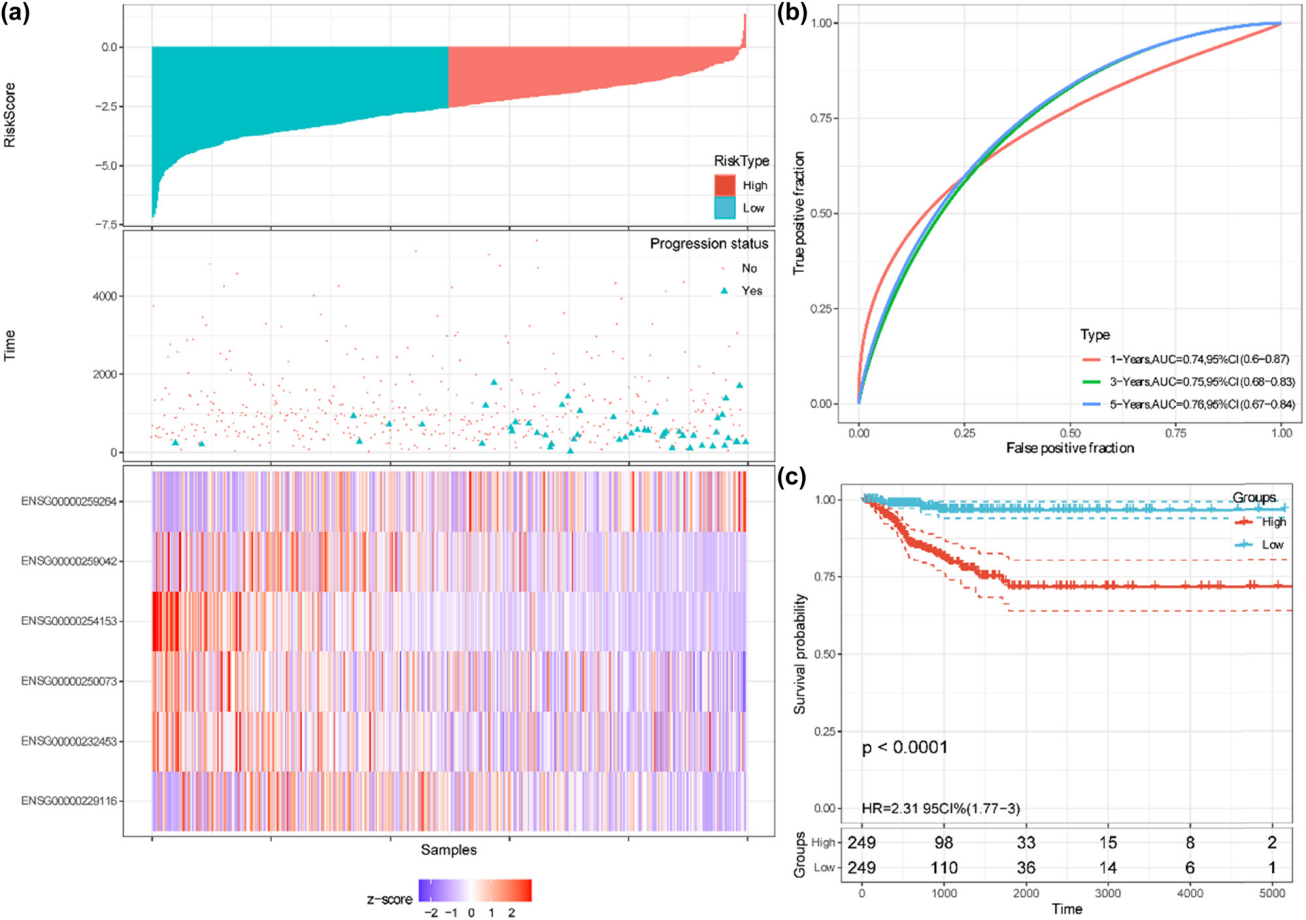
Predictive performance of the six autophagy-related lncRNAs signature in the entire cohort. (a) Risk scores, PFI/progression status, and expression heatmap. (b) Time-dependent ROC curves for predicting 1-, 3-, and 5-year PFI based on the risk scores. (c) Kaplan–Meier curves of the patients with high and low-risk scores, which were divided by the median value of the risk scores.

The prognostic performance of the autophagy-related lncRNAs risk model was further assessed by Kaplan–Meier plotting curves and time-dependent ROC curves in the training, validation, and entire cohorts. The patients in these cohorts were split into high-risk and low-risk groups with the median value of the risk scores. In the training cohort, the risk curve and the scatterplot showed that the low-risk group had lower risk scores and progression rates (Figure 5a). The corresponding expression profiles of the six autophagy-related lncRNAs were also visualized by heatmap (Figure 5a). The AUCs of 1-, 3-, and 5-year PFI were 0.78, 0.79, and 0.76, respectively (Figure 5b). Kaplan–Meier curve indicated that patients with low risk exerted more favorable PFI than those with high risk (*P* < 0.0001, Figure 5c). In the validation cohort, the risk score distribution, progression status, and the corresponding expression profiles of the six lncRNAs were also determined (Figure 6a). The AUCs of 1-, 3-, and 5-year PFI were 0.64, 0.71, and 0.79, respectively (Figure 6b). Kaplan–Meier curve showed that patients with low risk exerted more favorable PFI than those with high risk (*P* = 0.0033, Figure 6c). Similar results were identified in the entire cohort (Figure 7). The Harrell’s C-indices (95% CIs) of the six lncRNAs risk signature were 0.776 (0.692, 0.861), 0.717 (0.593, 0.841), and 0.756 (0.686, 0.825) in the training, validation, and entire cohorts, respectively. The predictive performance of the lncRNAs risk model was also compared with TNM stages, ATA risk stratification, and MACIS scores. In the patients without missing values (*n* = 480), Harrell’s C-index of the six lncRNAs signature was 0.759 and bigger than that of TNM stages, ATA risk stratification, and MACIS scores, whose C-indices were 0.631, 0.651, and 0.646, respectively (all *P* < 0.05; Table S1). Collectively, these results revealed that the risk signature exhibited good performance to predict the PFI of the PTC patients, and was superior to TNM stages, ATA risk stratification, and MACIS scores.

Finally, multivariate analysis results suggested that, whether in the training, validation, or entire cohorts, the six-lncRNAs risk scores (serve as a continuous variable) were an independent prognostic factor (Table 3). When the risk scores were equally split into two groups or three groups, high-risk scores were also identified as an independent prognostic factor.

**Table 3:** Univariate/multivariate COX regression analyses of the associations of autophagy-related lncRNAs risk scores with the PFI of patients with papillary thyroid carcinoma

Cohort/subgroups	Non-adjusted		Adjust I		Adjust II	
	HR (95% CI)	*P*-value	HR (95% CI)	*P*-value	HR (95% CI)	*P*-value
**Risk scores as a continuous variable**						
Training cohort	2.706 (1.935, 3.783)	<0.001	2.532 (1.690, 3.792)	<0.001	2.544 (1.699, 3.807)	<0.001
Validation cohort	1.794 (1.195, 2.694)	0.005	2.125 (1.157, 3.902)	0.015	2.575 (1.325, 5.004)	0.005
Entire cohort	2.303 (1.768, 2.999)	<0.001	2.082 (1.552, 2.794)	<0.001	2.128 (1.589, 2.851)	<0.001
**Risk scores as a categorical variable (two groups)**						
Training cohort						
Low	1		1		1	
High	11.285 (3.449, 36.923)	<0.001	9.191 (2.685, 31.469)	<0.001	10.898 (3.078, 38.581)	<0.001
Validation cohort						
Low	1		1		1	
High	5.233 (1.503, 18.218)	0.009	5.917 (1.375, 25.471)	0.017	9.547 (1.774, 51.381)	0.009
Entire cohort						
Low	1		1		1	
High	8.061 (3.439, 18.897)	<0.001	6.490 (2.694, 15.632)	<0.001	7.089 (2.917, 17.226)	<0.001
**Risk scores as a categorical variable (three groups)**						
Training cohort	1		1		1	
Low	1		1		1	
Medium	4.676 (1.010, 21.644)	0.049	4.683 (0.977, 22.454)	0.054	5.226 (1.063, 25.698)	0.042
High	12.279 (2.895, 52.088)	0.001	9.692 (2.116, 44.392)	0.003	11.256 (2.403, 52.714)	0.002
Validation cohort						
Low	1		1		1	
Medium	4.585 (0.512, 41.054)	0.173	4.130 (0.333, 51.205)	0.270	3.224 (0.261, 39.860)	0.362
High	13.257 (1.723, 101.986)	0.013	20.141 (1.777, 228.324)	0.015	34.465 (2.903, 409.094)	0.005
Entire cohort						
Low	1		1		1	
Medium	7.013 (1.582, 31.082)	0.010	6.771 (1.495, 30.673)	0.013	7.503 (1.629, 34.550)	0.010
High	20.216 (4.867, 83.973)	<0.001	16.905 (3.920, 72.898)	<0.001	19.531 (4.428, 86.148)	<0.001

Note: adjust I: adjust for age; histological types: TNM stage, T stage, N stage, M stage; tumor location: BRAF mutation and TERT mutation; adjust II: adjust for age, gender, race; histological types: TNM stage, T stage, N stage, M stage; tumor location: BRAF mutation and TERT mutation; HR, hazard ratio; CI, confidence interval.

### 3.4 Associations of the risk scores with clinical features and BRAF/TERT mutation status in PTC patients

**Figure 8:**
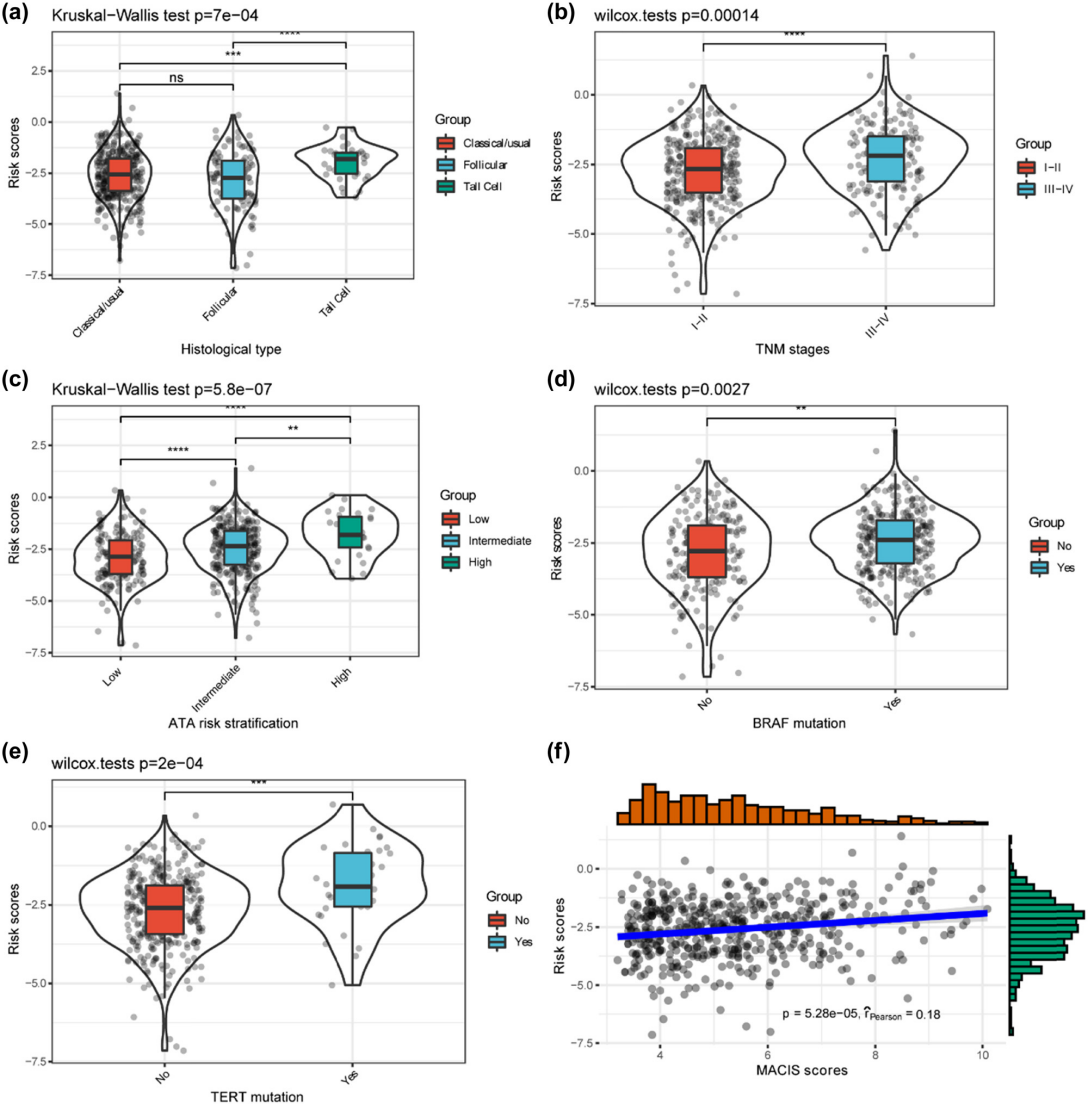
Comparison of the risk scores in patients with different features. (a) Comparison of the risk scores in patients with different histological types. (b) Comparison of the risk scores in patients with different TNM stages. (c) Comparison of the risk scores in patients with different ATA risk stratification. (d) Comparison of the risk scores in patients with or without BRAF mutation. (e) Comparison of the risk scores in patients with or without TERT mutation (f) Correlation of the risk scores and MACIS scores. Note: ns, not significant; **, *P* < 0.01; ***, *P* < 0.001; ****, *P* < 0.0001.

The associations of the six-lncRNAs risk scores with patients’ clinical features and BRAF/TERT mutation status were analyzed in the entire cohort. Patients were divided into high and low-risk groups by the median value and the features in different risk groups were compared. The results suggested that the patients with high risk had a higher prevalence of tall cell carcinomas, advanced stages, high ATA risk stratification, and higher TERT promoter mutation rate (Table 4). Comparisons of the original risk scores in patients with different features suggested patients with tall cell carcinomas (Figure 8a), III–IV stages (Figure 8b), high ATA risk stratification (Figure 8c), mutated BRAF (Figure 8d), or mutated TERT promoter (Figure 8e) had higher risk scores. The lncRNAs signature risk scores were also found to be positively correlated with MACIS scores (Figure 8f).

**Table 4:** Comparison of the clinical characteristics between the patients with low and high-risk scores

LncRNA signature scores	Low	High	*P*-value
N	249	249	
Age (years)	47.0 ± 15.3	47.7 ± 16.3	0.622
MACIS scores (*N* = 486)	5.2 ± 1.4	5.6 ± 1.6	<0.001
**Gender**			0.614
Female	179 (71.9%)	184 (73.9%)	
Male	70 (28.1%)	65 (26.1%)	
**Race**			0.536
White	160 (64.3%)	169 (67.9%)	
Asian	29 (11.6%)	22 (8.8%)	
Others	12 (4.8%)	16 (6.4%)	
Unknown	48 (19.3%)	42 (16.9%)	
**Cancer history**			0.286
No	229 (92.0%)	235 (94.4%)	
Yes	20 (8.0%)	14 (5.6%)	
**Thyroid gland disorder history**			0.174
No	128 (51.4%)	147 (59.0%)	
Yes	92 (36.9%)	73 (29.3%)	
Unknown	29 (11.6%)	29 (11.6%)	
**Histological types**			0.009
Classical/usual	181 (72.7%)	171 (68.7%)	
Follicular	56 (22.5%)	45 (18.1%)	
Tall cell	9 (3.6%)	27 (10.8%)	
Others	3 (1.2%)	6 (2.4%)	
**TNM stage**			0.022
Stage I	152 (61.0%)	127 (51.0%)	
Stage II	27 (10.8%)	25 (10.0%)	
Stage III	50 (20.1%)	61 (24.5%)	
Stage IV	18 (7.2%)	36 (14.5%)	
Unknown	2 (0.8%)	0 (0.0%)	
**T stage**			<0.001
T1	96 (38.6%)	46 (18.5%)	
T2	79 (31.7%)	83 (33.3%)	
T3	68 (27.3%)	102 (41.0%)	
T4	5 (2.0%)	17 (6.8%)	
TX	1 (0.4%)	1 (0.4%)	
**N stage**			0.062
N0	124 (49.8%)	104 (41.8%)	
N1	97 (39.0%)	123 (49.4%)	
NX	28 (11.2%)	22 (8.8%)	
**M stage**			0.281
M0	141 (56.6%)	140 (56.2%)	
M1	2 (0.8%)	7 (2.8%)	
MX	105 (42.2%)	102 (41.0%)	
Unknown	1 (0.4%)	0 (0.0%)	
**Tumor location**			0.005
Left lobe	96 (38.6%)	78 (31.3%)	
Right lobe	100 (40.2%)	110 (44.2%)	
Bilateral	47 (18.9%)	39 (15.7%)	
Isthmus	3 (1.2%)	19 (7.6%)	
Unknown	3 (1.2%)	3 (1.2%)	
**Residual tumor**			0.253
R0	195 (78.3%)	185 (74.3%)	
R1	19 (7.6%)	33 (13.3%)	
R2	2 (0.8%)	2 (0.8%)	
RX	18 (7.2%)	12 (4.8%)	
Unknown	15 (6.0%)	17 (6.8%)	
**ATA risk stratification**			<0.001
Low	108 (43.4%)	62 (24.9%)	
Intermediate	132 (53.0%)	159 (63.9%)	
High	6 (2.4%)	22 (8.8%)	
Unknown	3 (1.2%)	6 (2.4%)	
**BRAF mutation**			0.157
No	109 (43.8%)	88 (35.3%)	
Yes	133 (53.4%)	153 (61.4%)	
Unknown	7 (2.8%)	8 (3.2%)	
**TERT promoter mutation**			0.017
No	179 (71.9%)	161 (64.7%)	
Yes	10 (4.0%)	26 (10.4%)	
Unknown	60 (24.1%)	62 (24.9%)	

Note: ATA, American Thyroid Association.

### 3.5 Association of the risk scores with I-131 therapy benefits

**Figure 9:**
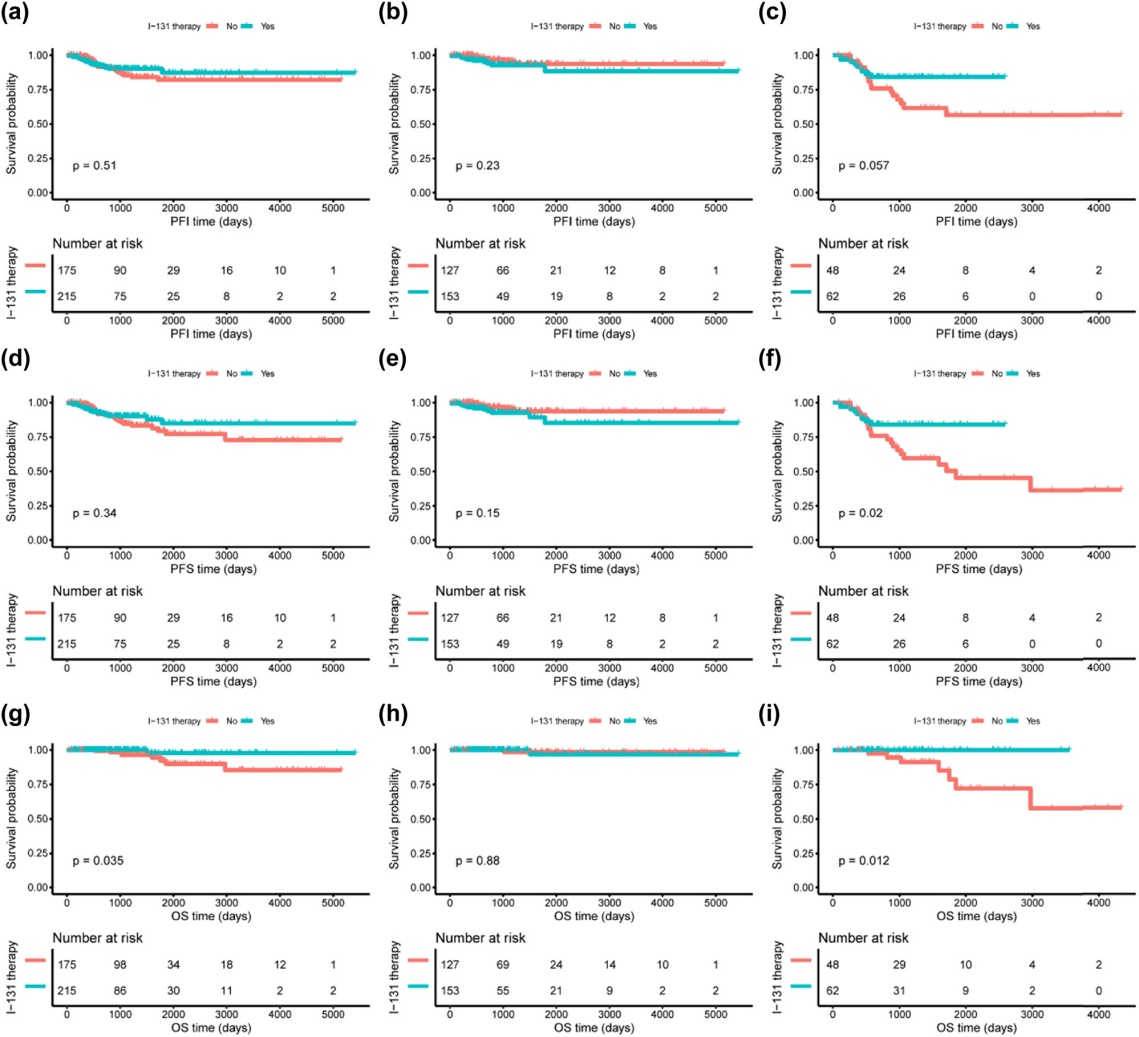
Comparisons of PFI, PFS, and OS between PTC patients with and without postoperative I-131 therapy in whole cohort or subgroups stratified by the risk scores. (a–c) Effects of I-131 therapy on PFI in the whole cohort (a), unfavorable subgroup (b), and favorable subgroup (c). (d–f) Effects of I-131 therapy on PFS in the whole cohort (d), unfavorable subgroup (e), and favorable subgroup (f). (g–i) Effects of I-131 therapy on OS in the whole cohort (g), unfavorable subgroup (h), and favorable subgroup (i).

Of the 390 patients, 215 (60%) with treatment information received postoperative I-131 therapy. No significant association of I-131 therapy with patients’ PFI was identified (*P* = 0.51; Figure 9a). However, when the patients were equally split into three groups by the LncRNAs signature risk scores, we observed a trend that I-131 therapy was associated with favorable PFI in the high-risk group while being associated with poor PFI in the low-risk group (data not shown), suggesting that the patients with high-risk scores but not those with low-risk scores might get benefit from I-131 therapy. To identify the patients favorable to I-131 therapy, the patients were divided into two groups (unfavorable group and favorable group) by a series of cutoff values, and the optimal cutoff value was selected by minimal *P*-value for interaction tests in univariate Cox analyses. One hundred ten (28%) and 280 (72%) patients in favorable (with high-risk scores) and unfavorable (with low-risk scores) groups were identified, respectively. As expected, I-131 therapy was associated with poor PFI in patients from the unfavorable group (*P* = 0.23; Figure 9b) while being associated with improved PFI in patients from the favorable group (*P* = 0.057; Figure 9c) although the associations did not reach statistical significance. We also used PFS and OS as secondary endpoints to explore the effects of the risk groups on I-131 therapy benefits. Although improved PFS was observed after I-131 therapy in whole 390 patients, the PFS difference was not significant (*P* = 0.34; Figure 9d). Similarly, a trend of an association between I-131 therapy and poor PFS was found in the unfavorable group (*P* = 0.15; Figure 9e) and I-131 therapy was significantly associated with improved PFS in the favorable group (*P* = 0.02; Figure 9f). As for OS (Figure 9g–i), I-131 therapy exerted no effects on OS in patients from the unfavorable group (*P* = 0.88; Figure 9h). In the favorable group, no death occurred in patients with I-131 therapy (*P* = 0.012; Figure 9i).

### 3.6 GSEA

**Figure 10:**
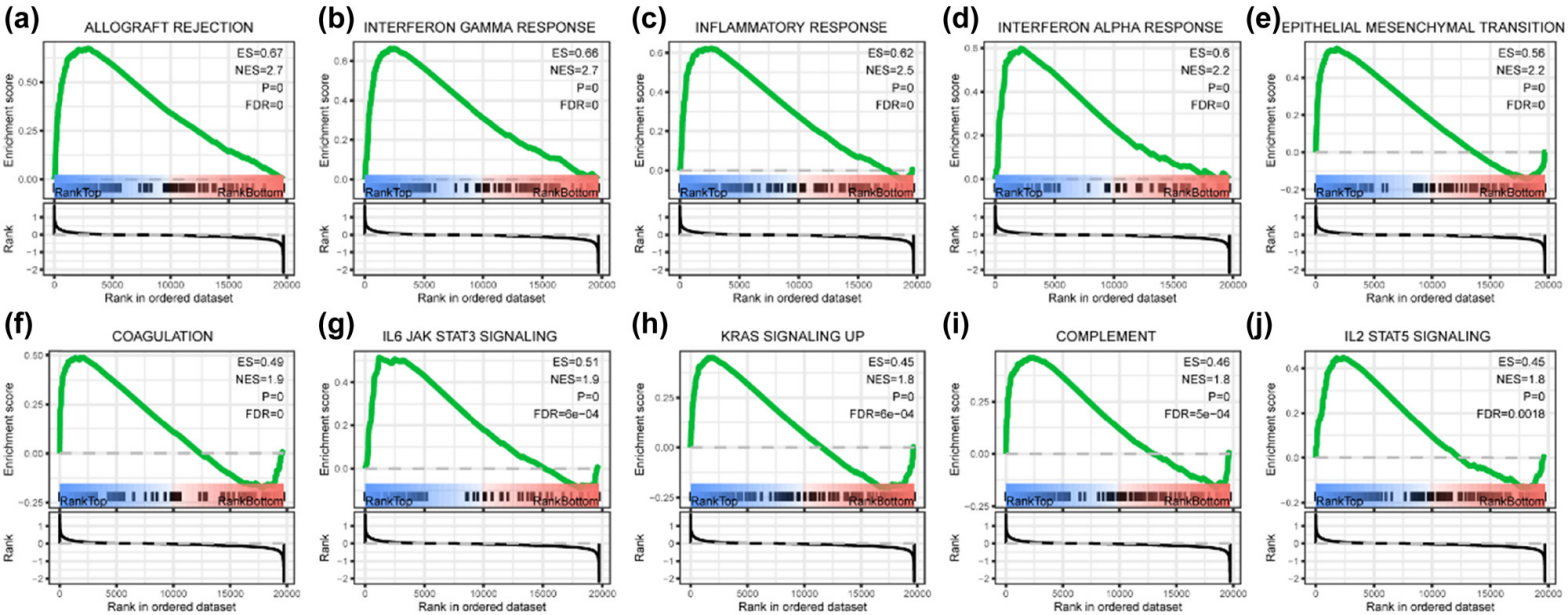
Top enriched hallmark gene sets between high and low-risk groups determined by GSEA: (a) allograft rejection, (b) interferon-gamma response, (c) inflammatory response, (d) interferon-alpha response, (e) epithelial–mesenchymal transition, (f) coagulation, (g) IL6 JAK STAT3 signaling, (h) Kras signaling up, (i) complement, and (j) IL2 STAT5 signaling.

GSEA was conducted to identify the differentially enriched hallmark gene sets between the high-risk (upper tertile) and low-risk (lower tertile) groups. The gene expression profiles of the high-risk and low-risk groups were compared and subjected to GSEA against hallmark gene sets. The results revealed that 21 gene sets were enriched in the high-risk group while none were enriched in the low-risk group based on the cut criteria. The top ten enriched gene sets in the high-risk group were allograft rejection, interferon-gamma response, inflammatory response, interferon-alpha response, epithelial–mesenchymal transition, coagulation, IL6 JAK STAT3 signaling, Kras signaling up, complement, and IL2 STAT5 signaling (Figure 10).

### 3.7 Exploration of the lncRNAs in PTC at a single-cell level

**Figure 11:**
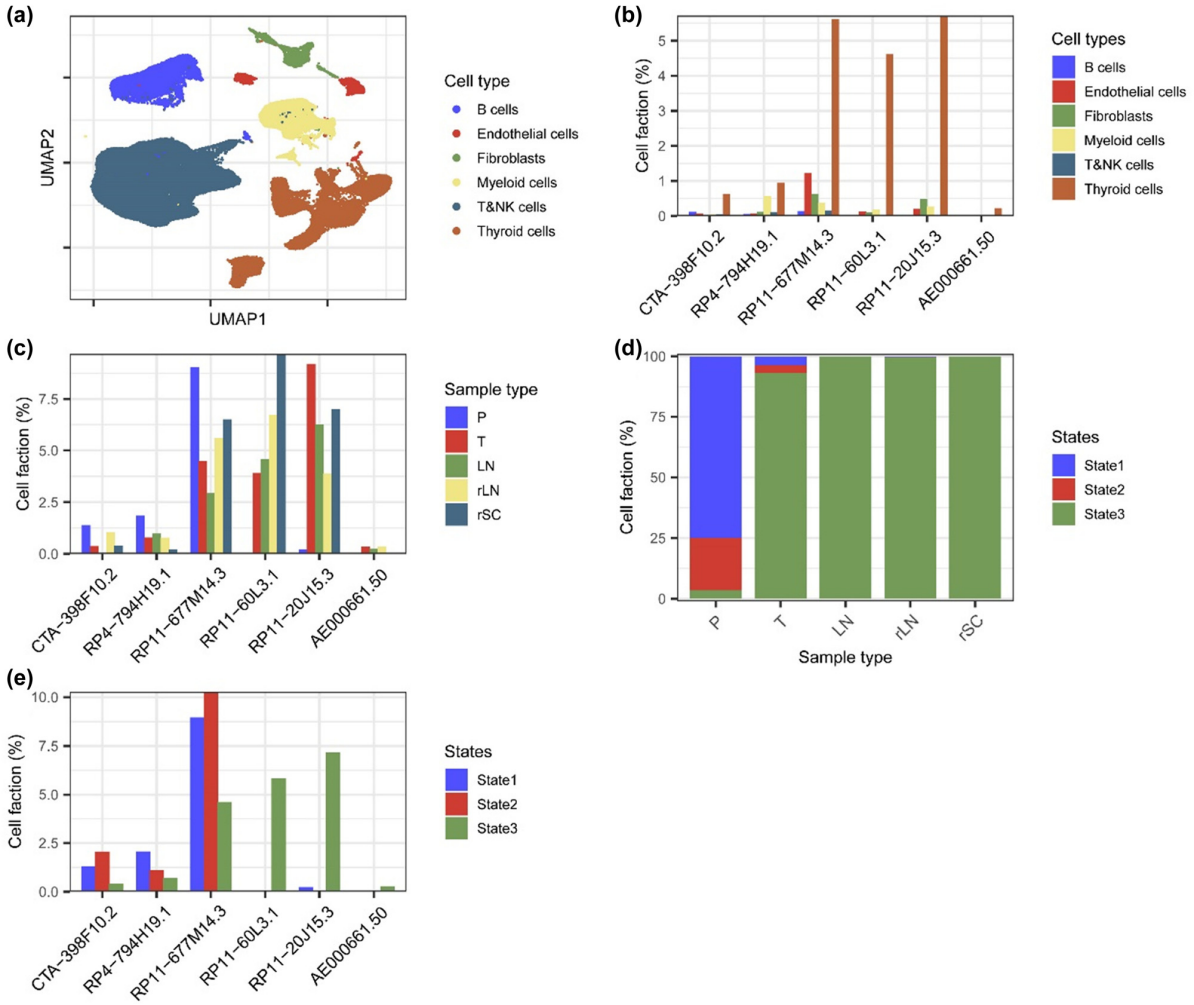
Exploration of the lncRNAs in papillary thyroid carcinoma at a single cell level. Single-cell RNA sequencing data of 22 samples from PTC patients were obtained from the GSE184362 dataset. (a) Cells were clustered into six main types and visualized by UMAP plot. (b) Proportions of the cells with different lncRNAs expression in different cell clusters. (c) Proportions of the thyroid cells with different lncRNAs expression in different sample types. P, paired adjacent normal tissues; T, primary tumors; LN, initially treated involved lymph nodes; rLN, recurrent LN; rSC, recurrent subcutaneous metastases. (d) Proportions of the thyroid cells with different state statuses in different sample types. (e) Proportions of the thyroid cells with different lncRNAs expression in different state cells.

**Figure 12:**
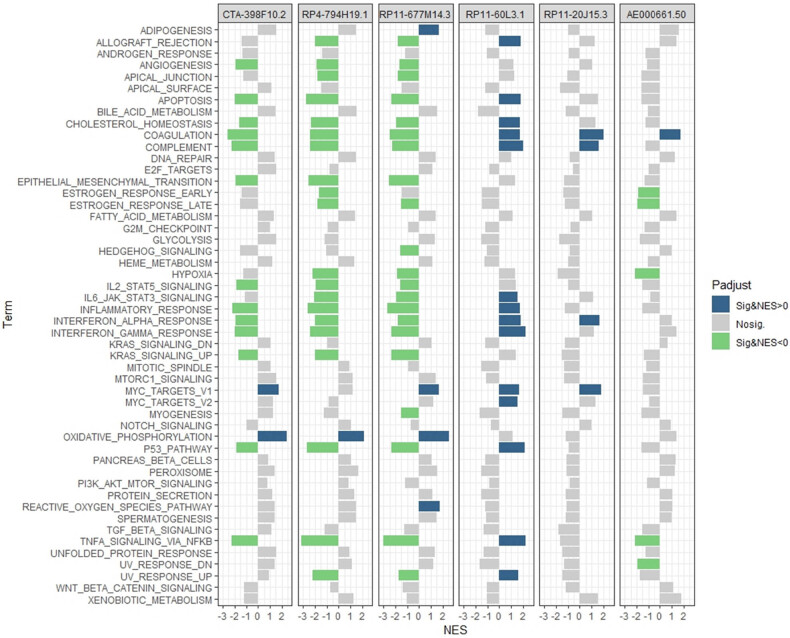
Hallmark pathways enrichment analysis between the thyroid cells with or without the corresponding LncRNA expression based on the single-cell RNA sequencing dataset GSE184362. NES, normalized enrichment score.

scRNA-seq data of 22 samples were extracted from the GEO database including six primary PTC tumors, six paired adjacent normal tissues, five initially treated involved LNs, three recurrent LNs, and two subcutaneous metastases. After quality control, a total of 156,295 cells remained for further analyses. Six main cell populations including B cells, endothelial cells, fibroblasts, myeloid cells, T/natural killer cells, and thyroid cells were identified according to corresponding markers (Figure 11a). The six lncRNAs in our signature were mainly expressed in thyroid cells (Figure 11b), then only thyroid cells were selected for subsequent analyses. The proportion of CTA-398F10.2, RP4-794H19.1, RP11-677M14.3 positive cells in tumor and LN samples was lower than that in adjacent normal samples while the proportion of RP11-60L3.1, RP11-20J15.3, and AE000661.50 positive cells was higher (Figure 11c), similar with the results from TCGA bulk RNA sequencing (Figure S1). The thyroid cells could be further clustered into nine subgroups, which included three developmental hierarchies (State 1–3) based on the cell markers derived from trajectory analysis in the previous report [27]. State 1 indicated the normal thyroid cells, state 2 indicated the premalignant cells, and state 3 indicated the malignant cells. Thyroid cells in adjacent normal samples were all with states 1 and 2 whereas cells with state 3 were enriched in the tumor, LN, and distance metastasis samples (Figure 11d). CTA-398F10.2, RP4-794H19.1, and RP11-677M14.3 were mainly expressed in states 1 and 2 cells while RP11-60L3.1, RP11-20J15.3, and AE000661.50 were mainly expressed in state 3 cells (Figure 11e). Furthermore, we performed hallmark pathway enrichment analyses between the thyroid cells with positive or negative LncRNA expression (Figure 12). A series of hallmark pathways were negatively associated with CTA-398F10.2, RP4-794H19.1, and RP11-677M14.3 expression, including angiogenesis, apoptosis, cholesterol homeostasis, coagulation, complement, epithelial–mesenchymal transition, IL2 STAT5 signaling, inflammatory response, interferon-alpha response, interferon-gamma response, Kras signaling up, P53 pathway, and TNFα signaling via NFκB. Most of those pathways were positively associated with RP11-60L3.1 expression. And only several pathways were significantly associated with RP11-20J15.3 and AE000661.50 expression.

## 4 Discussion

In the present study, we identified 199 autophagy-related lncRNAs in PTC and constructed a novel six-lncRNAs risk signature to predict patients’ PFI based on these lncRNAs. LncRNAs usually function by epigenetically regulated gene expression at different levels, including chromatin, gene splicing, transcription, and post-transcription. Although the function and clinical significance of the lncRNAs in thyroid cancer are not well understood, some of them have been identified to be involved in the autophagy process and thus affect the development and progression of thyroid cancer. Wang et al. have found lncRNA BANCR expression is upregulated in PTC and increases cell proliferation and activates autophagy [29]. Yang et al. have found that lncRNA TNRC6C-AS1 can downregulate STK4 methylation through the Hippo signaling pathway and then inhibit cell proliferation while promoting apoptosis and autophagy in thyroid cancer cells [22]. Zhao et al. have found that silencing lncRNA RP11-476D10.1 can inhibit cell proliferation while increasing apoptosis and autophagy of PTC cells through microRNA-138-5p-dependent inhibition of LRRK2 [30]. Gou et al. have found that lncRNA MALAT1 knockdown inhibits cell proliferation, migration, and invasion while increasing cell apoptosis and autophagy in thyroid cancer cells partly via the ceRNA network of MALAT1/miR-200a-3p/FOXA1 [23]. Gugnoni et al. have found that lncRNA LINC00941 can modulate cytoskeleton architecture and autophagy via regulating CDH6 in thyroid cancer cells [31]. Qin et al. have found that lncRNA GAS8-AS1, induced by ATF2, can promote autophagy by targeting miR-187-3p/ATG5 and miR-1343-3p/ATG7 axes in thyroid cancer cells [32]. Wen et al. have found that lncRNA SNHG9 inhibits cell autophagy whereas promotes cell apoptosis by YBOX3/P21 pathway in normal thyroid epithelial cells [33]. Peng et al. have found that lncRNA SLC26A4-AS1 overexpression can recruit ETS1 to promote ITPR1 expression and thereby promote autophagy and alleviate PTC progression [34]. In our study, we have identified 262 lncRNAs that were differently expressed in PTC compared to adjacent normal controls, and among them, 199 lncRNAs were negatively or positively correlated with ARGs (*R* ^2^ > 0.25). Then we utilized a series of methods, including univariate, LASSO, and stepwise multivariate COX regression analyses to establish a prognostic model based on the autophagy-related lncRNAs to predict PFI of PTC patients. The prognostic model consisted of six lncRNAs including CTA-398F10.2, RP4-794H19.1, RP11-677M14.3, RP11-60L3.1, RP11-20J15.3, and AE000661.50. The model was superior to TNM stages, ATA risk stratification, MACIS scores, and the previous model constructed with the key differentially expressed mRNAs regulated by differentially expressed circular RNAs [35]. In addition, we found that postoperative I-131 therapy was associated with favorable PFI, PFS, and OS in patients with high lncRNAs signature risk scores but not in those with low-risk scores, suggesting that the risk scores might be used to identify the patients who benefited from I-131 therapy and reduce unnecessary I-131 administration. Currently, the functions of our included lncRNAs in cancer were not clear. Gong et al. have found that CTA-398F10.2 can be increased by radiation in glioma cells but not in normal astrocytes [36]. Li et al. have found that the proto-oncogene JUN is correlated with RP4-794H19.1 and contributes to TNF signaling pathway in nasopharyngeal cancer [37]. James et al. have found that RP11-677M14.3 is associated with the different molecular subtypes of B cell acute lymphoblastic leukemia and co-occurrent with TGFB2 expression [38]. Liu et al. have mined data from 239 bladder cancer patients from TCGA database and constructed a multidimensional transcriptome signature including RP11-60L3.1 to predict the patient’s prognosis [39]. The molecular mechanisms of these lncRNAs in thyroid cancer need further investigation. By analyzing the single-cell RNA sequencing data, we found that the six lncRNAs were mainly expressed in thyroid cells but not stromal cells. Specifically, CTA-398F10.2, RP4-794H19.1, and RP11-677M14.3 were mainly expressed in normal and premalignant thyroid cells while RP11-60L3.1, RP11-20J15.3, and AE000661.50 were mainly expressed in malignant thyroid cells.

This is the first study to construct an autophagy-related lncRNAs signature, which shows a favorable prognostic performance. Nonetheless, there are still several limitations in the present study. First, the prognostic model was constructed based only on the data from TCGA database, and external validation with independent cohorts is needed. Second, further *in vitro* and *in vivo* research should be performed to investigate the molecular mechanisms and interrelation of these six lncRNAs in thyroid cancer.

## 5 Conclusion

In the present study, we identified 199 autophagy-related lncRNAs in PTC. Based on these lncRNAs, we constructed a novel six lncRNAs risk signature to predict the PFI of PTC patients, which exerts a good predictive performance in the training cohort as well as the validation cohort and is superior to TNM stages, ATA risk stratification, and MACIS scores.

## Supplementary Material

Supplementary material
