# Supplementary material for: A novel autophagy-related long non-coding RNAs signature predicting progression-free interval and I-131 therapy benefits in papillary thyroid carcinoma
Source: Open Med (Wars). 2023 Mar 3;18(1):20230660. doi: 10.1515/med-2023-0660 (PMC9985460; doi:10.1515/med-2023-0660)
Supplement: Supplementary material [file med-2023-0660-sm.pdf]

Supplementary material

Assessment of the tumors with ATA risk stratification and MACIS scores

Information on American Thyroid Association (ATA) risk stratification and MACIS scores of the PTC patients from TCGA database can be obtained from https://www.cbioportal.org/ or the related publication [1]. According to the related publication, the ATA stratification was performed based on the 2009 American Thyroid Association guidelines [2]. The metastasis, age, completeness of resection, invasion, and size (MACIS) score system is established by Hay et al. [3]. Criteria of ATA risk stratification and MACIS scores are as follows: (Figure S1) (Table S1)

ATA risk stratification

High risk

T4/T4a/T4b;

or M1.

Intermediate risk

T1/T2 and N1/N1a/ N1b;

or T3;

or tall cell subtype, but not T4/T4a/T4b or M1.

Low risk

T1/T2, NO/NX, and resection of R0/R1, but not tall cell subtype or columnar cell variants.

MACIS scores are based on the following variables

1. Distant Metastasis: M0 or MX, no distant metastases; M1, distant metastases present;
2. Age;
3. Complete Resection: R0 or RX, completely resected; R1 or R2, incompletely resected;
4. Local Invasion: None, no local invasion; T3 or T4a, local invasion;
5. Diameter of Tumor: Maximum value of dimension size.

The formula of MACIS scores: MACIS= 3.1 (if age <= 39 years) or 0.08 × age (if age >= 40 years), + 0.3 × tumor size (in centimeters), +1 (if incompletely resected), +1 (if locally invasive), +3 (if distant metastases present).

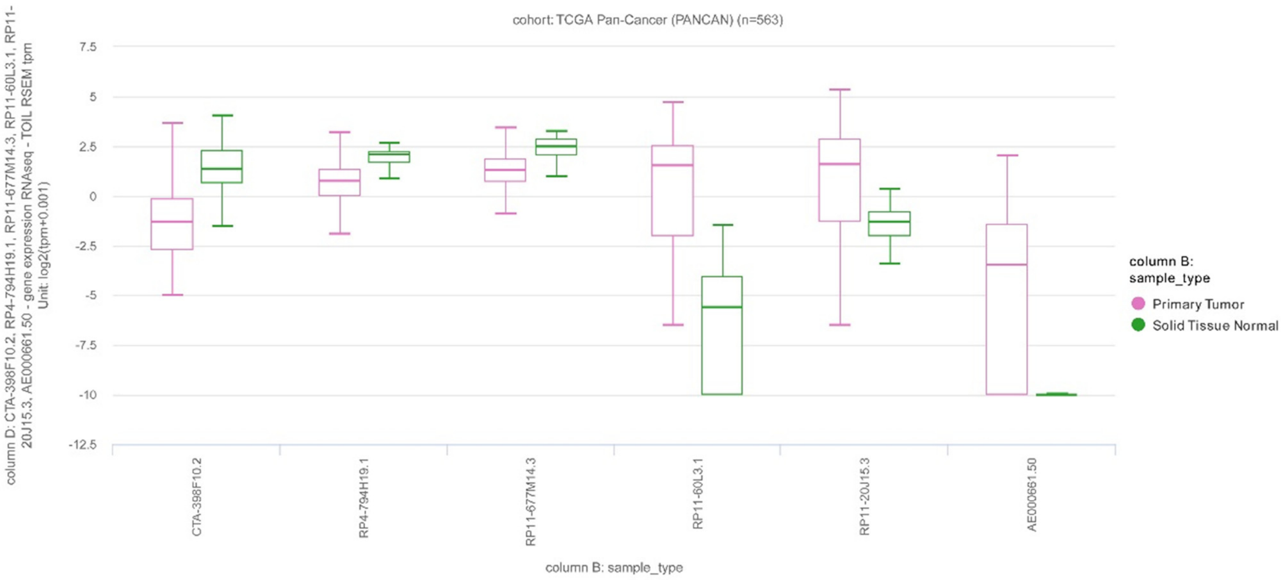

Figure S1: Expression of CTA-398F10.2, RP4-794H19.1, RP11-677M14.3, RP11-60L3.1, RP11-20J15.3, and AE000661.50 in primary thyroid tumor samples and adjacent normal tissues (solid tissue normal) samples. The results were based on the data from TCGA and visualized by the UCSC Xena web tool (xenabrowser.net).

Table S1: Comparison of Harrell's C-indices of the lncRNA signature with TNM stages, ATA risk stratification, and MACIS scores in predicting progression-free interval of patients with papillary thyroid carcinoma

Predictors	Harrell's C index (95%CI)	P-value
LncRNA signature	0.759 (0.688, 0.829)	Reference
TNM stage	0.631 (0.552, 0.710)	0.013
ATA risk stratification	0.651 (0.591, 0.710)	0.015
MACIS scores	0.646 (0.567, 0.726)	0.040

References

[1] Cancer Genome Atlas Research N. Integrated genomic characterization of papillary thyroid carcinoma. Cell. 2014;159(3):676–90.
[2] Cooper DS, Doherty GM, Haugen BR, Kloos RT, Lee SL, Mandel SJ, et al. Revised American Thyroid Association management guidelines for patients with thyroid nodules and differentiated thyroid cancer: the American Thyroid Association (ATA) guidelines taskforce on thyroid nodules and differentiated thyroid cancer. Thyroid. 2009;19(11):1167–214.
[3] Hay ID, Bergstralh EJ, Goellner JR, Ebersold JR, Grant CS. Predicting outcome in papillary thyroid carcinoma: Development of a reliable prognostic scoring system in a cohort of 1779 patients surgically treated at one institution during 1940 through 1989. Surgery. 1993;114(6):1050–8.
